# Potential therapeutic targets in the tumor microenvironment of hepatocellular carcinoma: reversing the protumor effect of tumor-associated macrophages

**DOI:** 10.1186/s13046-021-01873-2

**Published:** 2021-02-17

**Authors:** Jingyi Zhou, Weiyu Wang, Qi Li

**Affiliations:** Department of Oncology, Shanghai General Hospital, Shanghai Jiao Tong University School of Medicine, 100 Haining Road, Shanghai, 200080 People’s Republic of China

**Keywords:** Tumor microenvironment, Hepatocellular carcinoma, Tumor-associated macrophages, Immunotherapy, Immune checkpoint, Antitumor immunity

## Abstract

In hepatocellular carcinoma patients, due to the microenvironmental specificity of liver, the tumor microenvironment exhibits high immunosuppression and drug resistance, resulting in excessive or insufficient responses to immunotherapy. The dynamic interactions between tumor cells and immune modulators in the TME significantly impact the occurrence and development of tumors, efficacy, and drug resistance, which can create a much more positive response to immunotherapy. Moreover, with the wide application of single-cell sequencing technology in the TME, increasing evidence shows an interaction network among cells. Sequencing results suggest that specific tumor-associated macrophages are a hub node, connecting different cell populations in the cell interaction network, and can could regulate tumor generation and antitumor immunity. This review focused on therapeutic targets that could be targeted to remodel the tumor microenvironment and reprogram the tumor-associated macrophage phenotype in hepatocellular carcinoma patients, thereby improving immunotherapeutic efficacy.

## Background

Hepatocellular carcinoma (HCC), accounting for more than 80% of primary liver cancer cases [1], is one of the most common malignant solid tumors and the fourth most frequent cause of cancer-related mortality worldwide [2]. Furthermore, the international trend for liver cancer incidence is still grim. The incidence of liver cancer continues to increase and is rising more rapidly than that of other types of cancer. From 2007 through 2016, the rate of increase was approximately 2 to 3% annually. According to cancer statistics, in 2020, 42,810 new cases of liver and intrahepatic bile duct cancers and 30,160 liver cancer-related deaths occurred in the United States [3]. The most recent liver cancer statistical data in China estimated 370,000 new cases (274,000 males and 96,000 females) of liver cancer in 2015. For liver cancer-related mortality, there were 326,000 new deaths (242,000 males and 84,000 females) in China [4]. As the predominant form of liver cancer, HCC tends to occur in patients with a history of cirrhosis, with these patients accounting for more than 70% of the HCC patients population [5]. Major risk factors that contribute to fibrosis and cirrhosis include chronic hepatitis B virus (HBV) infection, chronic hepatitis C virus (HCV) infection, obesity, diabetes, excess alcohol consumption, and metabolic diseases [6, 7]. In the setting of cirrhosis, genetic mutations, epigenetic deregulation, and abnormal molecular signaling pathway transduction are the most common causes of hepatocellular carcinogenesis [8].

With the deepening of tumor immunology research, studies have revealed that the tumor microenvironment (TME) is an intricate ecosystem around cancer cells that supports carcinogenesis from cancer initiation to metastasis and is consistently modulated by cellular metabolism, genetic alterations, dysfunctional oncogenic signaling, and epigenetic factors. The first discovery of the link between chronic inflammation and oncogenesis and the observation of leukocytes in neoplastic tissues by Rudolf Virchow indicated the need to develop a broader understanding of the TME in solid malignant tissue [9]. The skeleton of this complex tissue microenvironment is built by the extracellular matrix (ECM) and vascular networks, and the gaps are filled with different kinds of cells. In addition to tumor cells, many types of stromal cells, fibroblast, adaptive and innate immune cells, and noncellular components, such as cytokines, signaling proteins, and growth factors, are found in the TME [10]. Furthermore, researchers have verified that intratumoral heterogeneity is significantly related to different immune microenvironments [11], which dramatically influences precision medicine and will be elaborated later.

More specifically, the tumor immune microenvironment (TIME), the stage for the interactions between tumor cells and immune cells, plays a fundamental and indispensable role in HCC evolution and greatly influences immunotherapy outcomes. Within the TIME, many immune cells have been found to accumulate during tumor progression, such as myeloid-derived suppressor cells (MDSCs), regulatory T (Treg) cells, and tumor-associated macrophages (TAMs), which are responsible for building of immunosuppressive environment. In contrast, natural killer (NK) cells, cytotoxic CD8^+^ T cells, and CD4^+^ T cells with a proinflammatory T helper 1 phenotype work together to counteract protumor effects. Recently, according to the degree of immune infiltrate, Mikhail Binnewies et al. divided the TIME into three classes: infiltrated-excluded (I-E) TIMEs, infiltrated-inflamed (I-I) TIMEs, and tertiary lymphoid structure (TLS) TIMEs [12]. I-E TIMEs are filled with immune cells but lack cytotoxic lymphocytes (CTLs) in the tumor core, while I-I TIMEs are significantly infiltrated with CTLs expressing programmed cell death 1 (PD-1), and leukocytes and tumor cells within I-I TIMEs are characterized by the expression of immune-dampening PD-1 ligand (PD-L1). TLS-TIMEs are a subclass of I-I TIMEs and have been observed to include TLSs containing lymphoid aggregates; their cell composition is similar to that in the lymph nodes. These classifications of the immune contents within the TME are the leading information to recognize different the immunological compositions and immune statuses of tumors (i.e., activated or suppressed); for instance, tumors with an I-E TIME are known as immunologically “hot” tumors, which may produce positive responsiveness to immunotherapy and affect overall survival.

Generally, surgery (liver resection or transplantation) and radiofrequency ablation (RFA) are the standard curative therapies for HCC. In addition, transarterial chemoembolization (TACE) or transarterial radioembolization (TARE) are promising locoregional therapies that can reduce mortality and improve patient quality of life [13, 14]. For unresectable or advanced HCC, targeted molecular therapies, such as multikinase vascular endothelial growth factor (VEGF) inhibitors, are believed to be the future of HCC treatment. In the past decade, sorafenib has been the first-line agent most widely used for systemic chemotherapy [15]. As an FDA-approved tyrosine kinase inhibitor (TKI), sorafenib targets multiple receptors including platelet-derived growth factor (PDGFR) and fibroblast growth factor (FGFR1). It inhibits VEGF signaling, thereby downregulating the proliferation, migration, and angiogenesis of hepatocellular carcinoma cells and thus significantly prolonging HCC patient overall survival [16]. This year, a phase 3 trial enrolling patients with hepatocellular carcinoma showed that the immunotherapeutic combination of atezolizumab and bevacizumab produced a better outcome than sorafenib. With a 67.2% OS rate at 12 months and 6.8 months as the mPFS time, atezolizumab-bevacizumab was superior to sorafenib in terms of 12-month OS and PFS outcomes, revealing a promising therapeutic strategy for unresectable HCC patients [17, 18].

Given that the abovementioned curative therapies are only suitable for patients with a limited tumor burden, TIME-based approaches tailored to individual patients provide a prospective HCC treatment. This review focuses on the novel and innovative targets in the TME for immune checkpoint blockade (ICB) agents in HCC. In addition, since macrophage infiltration in the TME is a critical link in the development of liver cancer induced by steatosis, we discuss the potential of TAMs as targets for HCC immunotherapy. Furthermore, an inhibitor of colony-stimulating factor (CSF)-1 receptor targeting TAMs was shown to exert significant antitumor effects on the mouse glioblastoma and improve the survival rate [19]. We discuss emerging methods that could reprogram the tumor-associated macrophage phenotype and remodel the TME in HCC patients, thereby enhancing the antitumor immune response.

### Rationales for using the TME as a therapeutic basis in HCC

The HCC tumor immune microenvironment is involved in the infiltration of various innate and adaptive immune cells and the diverse expression of genes that affect cancer immune surveillance and the response to immunotherapy. Different tumor subtypes show different patterns for the immune microenvironment, which is partially caused by intratumoral heterogeneity. HCC and intrahepatic cholangiocarcinoma (iCCA) are the two clinically and pathologically distinct categories of liver cancer [20], gene expression analysis, single-cell RNA sequencing, flow cytometric analysis while through histological analysis, such as multiplex immunohistochemistry, and histological analysis, such as multiplex immunohistochemistry, [21], have shown that the cellular composition of the TIME varies significantly among different tumor subtypes [11, 22, 23]. Therefore, with reference to the classification of histopathological subtypes according to the characteristics of the stromal features of HCC (e.g., lymphocyte-rich HCC, sclerosing HCC, and steatohepatitis HCC) [24, 25], a comprehensive evaluation of the immune microenvironment could serve as a classification standard and benefit precision medicine by stratifying the target population for immunotherapy.

### Characteristics of the TIME in HCC

**Fig. 1:**
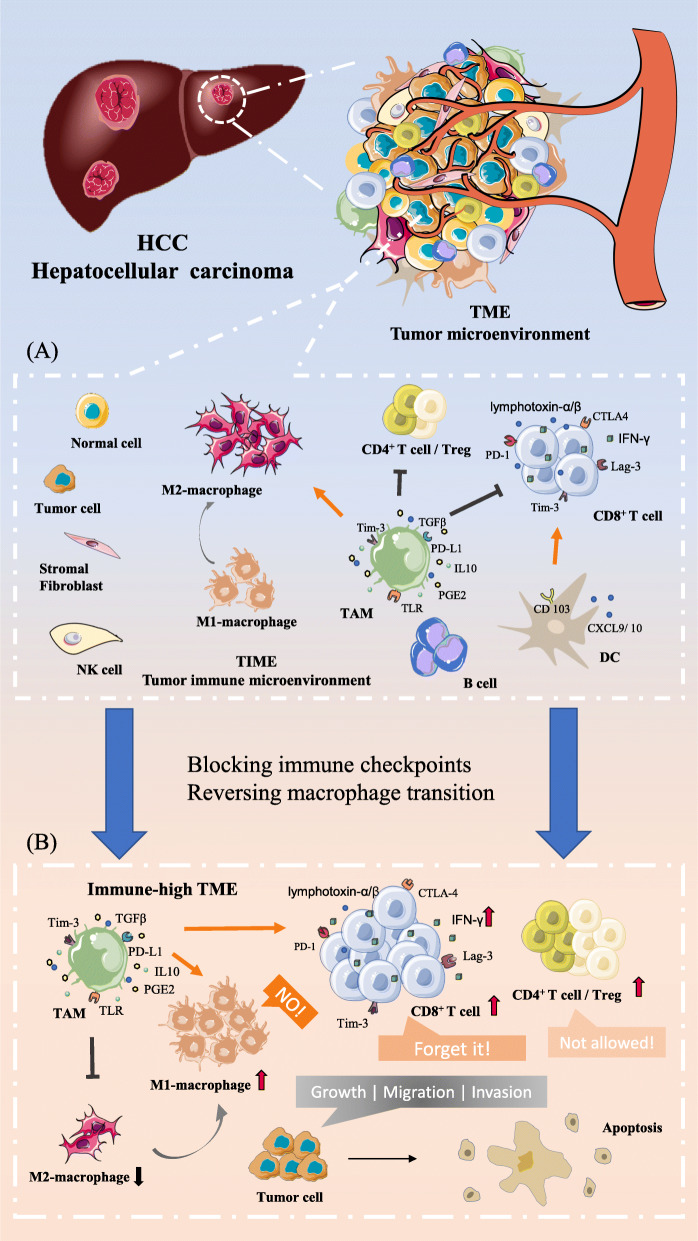
Dynamic changes in cellular components in the HCC TME. **a** According to cell functions in the TME, the TME can be refined into the tumor immune microenvironment (TIME). Due to the specific features of the liver, the role of tumor-associated macrophages (TAMs) in the liver TIME is prominent. As shown, during the development of HCC, the expression of immunosuppressive checkpoint molecules (Tim-3, PD-1, and TLR) on the surface of TAMs affects the accumulation and antigen presentation of cell performing immune surveillance. **b** Blocking immune checkpoint molecule expression and reversing the phenotype of macrophages are two main approaches to regulate the tumor immune microenvironment discussed in this review. As shown above, increased infiltration of T cells and M2 macrophages can effectively inhibit the growth, metastasis, and invasion of tumor cells at the cellular level, which in turn achieves superior immunotherapeutic efficacy

In the tumor immune microenvironment, the composition of immune cells (including most innate and adaptive immune cells) is roughly the same, but the degree of infiltration of different immune cells varies greatly, as shown in Fig. 1(a). For prognosis, after surgical HCC resection, a higher CD8^+^ T cell/Treg ratio is essential for effective antitumor immunity [26]. Moreover, ectopic lymphoid follicles, highly ordered structures formed by immune cells, rarely occur in HCC [27]. Through estimating immune cell infiltration, researchers have divided HCC into immune-class HCC and exhausted-class HCC [23, 28]. Immune-class HCC is characterized by high expression of T cell and B cell genes, interferon (IFN)-related genes and PD-1/PD-L1. Furthermore, in active-immune-class HCC, in addition to the elevated expression of the listed genes, the fibroblast-related gene signature is significantly lower than that in exhausted-class HCC [29]. Furthermore, histopathologically, according to T cell, B cell, and lymphoplasmacytic infiltration, immunity in the HCC microenvironment can be divided into three levels: immune-high, immune-mid, and immune-low. High co-infiltration of immune cells is the feature of the immune-high subtype associated with a relatively good prognosis, while the immune-mid and immune-low subtypes lack B cells, plasma cells and other immune cells. Although the immune-high subtype of HCC, which shows high expression of IFN-related genes and PD-1/PD-L1 molecules similar to immune-class HCC [23], represents active antitumor immunity, this subtype has been observed in poorly differentiated HCC with a poor prognosis [30, 31]. This finding suggests that it is reasonable to evaluate the TIME as one of the criteria for judging the prognosis of HCC.

In addition, as a classic multistage carcinogenesis model, the TIME of HCC also constantly changes during tumor progression. From low-grade dysplastic nodules (LGDNs) to high-grade dysplastic nodules (HGDNs), B cell infiltration and lymphoid follicle formation increase significantly but then decrease during the progression from early HCC to advanced HCC [32]. Moreover, from moderately differentiated lesions to poorly differentiated lesions, the number of Treg cells increases, while the infiltration of NK cells and macrophages and the CD8^+^ /CD4^+^ T cells ratio decrease [33].

### Pro- and antitumor effect of immune cells in the TIME of HCC

As an immunomodulatory organ under disease-free conditions, the liver is responsible for processing multiple antigens derived from the gut and portal vein blood circulation via its intrinsic immune tolerogenicity that inhibits inappropriate inflammatory responses. However, once this complex immune tolerance system is disrupted due to chronic inflammatory liver disease, the functions of innate and adaptive cells become dysregulated, and cancer cells escape immune surveillance, thereby facilitating liver tumor development [34, 35]. According to the cell differentiation source and function, immune cells in the TIME of HCC can be grouped into three categories: antitumor lymphocytes (CTLs and NK cells), immunosuppressive lymphocytes (Tregs), and immunosuppressive myeloid cells (TAMs, DCs, and MDSCs). Compared to the peripheral blood, the liver has a higher ratio of CD8^+^ T cells to CD4^+^ T cells [36]. However, CD8^+^ T lymphocytes, the tumor-infiltrating lymphocyte (TIL) subset known to be the primary antitumor effector population in liver cancer, also play a conflicting role in facilitating a chronic proinflammatory microenvironment and spontaneous immune surveillance. CD8^+^ CTLs directly contact and lyse cells via secretion of perforin, granzyme A, and granzyme B; they also mediate signal transduction through cell-surface Fas-Fas ligand interactions and secretion of IFN-γ and TNF [37].

On the one hand, CD8^+^ CTLs have been found to promote HCC development by producing lymphotoxin-α and lymphotoxin-β, which are related to the construction of ectopic lymphoid structures (one of the poor prognostic factors of HCC), and tumor progression was shown to be slowed down when CD8^+^ T cells and lymphotoxin-β were depleted in an HCC model. In comparison, the number of IFN-γ-secreting CD8^+^ T cells, which are responsible for cytotoxic interactions in HCC, is positively correlated with improved OS [38]. Similarly, in a specific HCC model involving overexpression of the hepatocyte-specific proto-oncoprotein c-Myc with a methionine and choline-inadequate diet, depletion of CD4^+^ T cells accelerated the development of HCC [39], but after HCC resection, tumor-infiltrating CD4^+^ CTLs are known to be independent predictors of disease-free survival. Treg cells infiltration has a negative correlation with the prognosis of HCC. Therefore, we may expect further study on the opposing role of different T cell subsets in different stages of HCC pathogenesis.

Although the function of T lymphocytes in the progression of HCC and that of TIME-infiltrating T cells remain controversial, communication between DCs and T cells is irreplaceable for in effective antitumor immunity. DCs are the professional antigen-presenting cells (APCs) that activate effector CD4^+^ and CD8^+^ T cells. One DC subtype, CD103^+^ DCs, is the primary source of the chemokines CXCL9 and CXCL10, two necessary chemokines responsible for recruiting CD8^+^ T cells into the TME. When CD103^+^ DCs are lacking in the tumor, T cell migration into the tumor is impaired, damaging antitumor responses [40]. Additionally, the largest population of resident macrophages (Kupffer cells (KCs)) in the liver constitutes another major APC population. By expressing scavenger receptors, Toll-like receptors (TLRs), and other receptors, KCs identify antigens and internalize pathogens and apoptotic cells. However, KC-induced antigen-presentation can also upregulate the expression of PD-L1 and the release of immunosuppressive molecules, such as IL-10, TGFβ, and prostaglandin E2 (PGE2), which result in the induction of immunosuppressive Tregs and facilitates T cell suppression. TAMs are also present [41]. According to an analysis of HCC data in The Cancer Genome Atlas (TCGA), high TAM infiltration influences patient survival and correlates with poor outcomes in HCC [42]. To investigate the underlying mechanisms of TAM-mediated pro-HCC effects, a recent study using human HCC samples identified the TAM phenotype CD14^+^HLA-DR^+^PD-L1^+^Lag-3^+^ [43]. Importantly, further research shows that T cell immunoglobulin mucin-3 (Tim-3) on macrophages promotes the differentiation of primitive macrophages into M2 macrophages, thereby upregulating the secretion of anti-inflammatory cytokines and enhancing IL-6-induced tumor growth in patients with HCC. Underlying this, transforming growth factor-β (TGF-β) promotes the transcription of Tim-3 in TAMs [44]. Furthermore, Jindao et al. recently found that M2 macrophage-derived exosomes promoted HCC cell migratory activity by transferring functional CD11b/CD18 proteins from TAMs to HCC cells [45]. Interestingly, researchers also found that miR-92a-2-5p in macrophage-derived exosomes could decrease androgen receptor expression, which results in HCC cell invasion [46].

It is well accepted that there is a high density of immune cells in the liver, regardless of whether they serve as immunosurveillance effectors or exert immunosuppressive effects; they are all components of the TIME in HCC and act as immunological modulators by responding to various cell-surface ligands, producing a wide array of pro- and anti-inflammatory molecules, or directly killing cancer cells to affect the activity of tumor cells. Therefore, therapies that reactivate surveillance-capable immune cells or keep immunosuppressive cells of the innate and adaptive immune systems dormant in the TIME may produce promising effects to treating patients with HCC.

### Immune checkpoint inhibition (ICI) for HCC treatment

It is well known that the tumor expression of immunoregulatory molecules may reflect the immune milieu, which is regulated by alterations in oncogenic cellular signals. Then, different expression levels of molecular subclasses and immune phenotypes lead to specific tumor immune microenvironments. Additionally, immune checkpoints are a group of signaling pathway molecules expressed by immune cells that regulate and control immune responses while maintaining self-tolerance, preventing the immune system from performing unregulated attacks on cells and preventing autoimmunity. Since the efficacy of immune responses is determined by the delicate balance between costimulatory and coinhibitory signals, immune checkpoints can be either stimulatory or inhibitory. For instance, stimulatory immune checkpoint molecules include CD27, CD28, CD40, CD122, and CD137. Inhibitory immune checkpoint molecules include A2AR, B7-H3, B7-H4, cytotoxic T-lymphocyte antigen 4 (CTLA-4), indoleamine 2,3-dioxygenase (IDO), lymphocyte activation gene 3 (LAG-3), PD-1, and Tim-3 [47–50]. However, immune checkpoints are usually “hijacked” by tumor cells to restrain the immune system’s ability to mount an effective antitumor response to protect the tumor from attack by the immune system. The expression of immune-checkpoint proteins, including CTLA-4, PD-1, PD-L1, and IDO-1, results in a protumor cytokine milieu and further moderates the antitumor response [51, 52].

Interestingly, Chew et al. found that compared with Tregs and T cells isolated from a non-TME location, Tregs and T cells within the TME expressed multiple markers responsible for T-cell exhaustion, including PD-1, Lag-3, and Tim-3 [43]. In addition, Brown et al. indicated that the high expression of IDO in patients with HCC is associated with tumor cell the resistance ICBs [53]. Therefore, the emerging goal of immunotherapy is to curb T cell depletion by tumor cells and increase the rate of active immune cell infiltration, thereby allowing detection and elimination of tumor cells and preventing further tumor development.

### Targets for ICBs in the HCC tumor microenvironment

Through targeting of receptor or ligand interactions by molecular blockade, ICB / ICI has become the first generation of antibody-based immunological treatments that target the immunoregulatory dampening of T cell activation or the function of host responses to tumor-associated antigens. The ability to induce tumor regression in murine models by blocking the prototypical receptor CTLA-4 was discovered over 20 years ago [54]. Since anti-CTLA-4 (ipilimumab) was approved for melanoma treatment, which promotes T-cell activation by blocking the interaction of CTLA4 with CD80 and CD86 [55], the clinical successes achieved by blocking CTLA-4 and PD-1 have inspired further exploration of the potential of ICB therapies. In addition to the anti-CTLA-4 antibody ipilimumab in melanoma, clinical studies of the anti-PD-1 antibody nivolumab in advanced HCC patients have also proven this antibody to be efficient in phase I/II clinical trials with satisfactory safety and response data [56–58]. However, there is still a large population of patients who show an ineffective response after application of ICB therapy.

Retrospective analyses of HCC patients treated with ICB have indicated that various TIME subtypes associated with tumor progression exhibit different ICB responsiveness patterns [12]. In HCC tissue, as components of the TIME, CD8^+^ and CD4^+^ T cells expresses a higher surface levels of PD-1, Tim-3, and CTLA-4 than those in the peripheral blood or nontumor tissues that express the ligands for these receptors [59]. After further evaluation, researchers determined that the expression of PD-1, Tim-3, and LAG-3 is higher on the cell surface of tumor-associated antigen (TAA)-specific CD8^+^ TILs than those on that of other CD8^+^ TILs. Once antibodies block PD-L1, Tim-3, or LAG-3, T cell responses to tumor antigens in HCC could be restored, and combination treatment effects would also be enhanced. Although ICB studies of anti-PD-1/PD-L1 and anti-CTLA-4 antibodies have significant advantages in multiple tumor treatments, there has been an increasing incidence of resistance to these antibodies in recent years. In addition, accumulating co-expression of PD-1 and Tim-3 on CD8^+^ T cells has been observed in the TME [60, 61], and studies have also revealed that blockade of PD-1 simultaneously increases the expression of other checkpoints molecules on immune cells in the TME, including Tim-3 and LAG-3 [62, 63]. Therefore, anti-Tim-3 antibodies have become a new focus of curative HCC research and have shown antitumor efficacy in some preclinical studies. In the TME of HCC, Tim-3 is expressed at high levels on tumor cells, tumor-infiltrating T cells, Tregs, and TAMs, inducing TAM-immunosuppression and promoting HCC progression by dampening T cell function. Consistently, Li et al. reported that the relatively high infiltration of Tim-3^+^ T cells in HCC tissues leads to shortened patient survival [61].

In addition to the prognostic characteristics of Tim-3 in HCC, several anti-Tim-3 antibodies have been granted research patents. In monotherapy or combination therapy, the anti-Tim3 antibody RMT3–23 exhibits significant antitumor efficacy in several types of cancer, such as MCA-induced sarcoma [64], colon adenocarcinoma [64], lung cancer [60], ID8 ovarian cancer [65], melanoma [63], and murine glioma [66]. However, triggering antitumor immunity in tumors requires the co-existence of CD4^+^ T cells and IFN-γ-expressing CD8^+^ T cells when using anti-Tim-3 antibodies as a monotherapy [64]. Moreover, for the treatment of some cancer types, the efficacy of a monotherapy is significantly inferior to that of multi-immune checkpoint-targeted combination therapy. Co-blockade of Tim-3, PD-1, and CTLA-4 or combination with other therapeutic strategies, such as DNA methyltransferase inhibition or stereotactic radiosurgery, has shown synergistic antitumor effects in preclinic studies [67]. Although researchers have revealed that directly blocking the expression of Tim-3 and PD-1 can restore the antitumor function of TILs in HBV-HCC [68], there are almost no relevant experimental data showing that targeted blockade of Tim-3 can inhibit the growth of HCC and improve the survival rate of patients. Based on the antitumor effects of Tim-3 in other cancer types, Tim-3 could be a promising target for novel immunotherapeutic approaches in HCC, as shown in Fig. 1(b).

### Potential regulatory strategy for ICI in HCC

Antibody application in phase III clinical trials assessing PD-1, and CTLA-4 ICB monotherapies has shown inspiring outcomes, but the production of monoclonal antibodies is difficult and costly; therefore, other forms of inhibitors or mechanistic regulation are alternative strategies.

MiRNAs, as dual regulators, are widely used in the study of tumorigenic mechanisms and therapeutic tools. Many reports have revealed that tumor-suppressing miRNAs are responsible for the antitumor immune response by directly regulating the transcription of immune checkpoint molecules, such as PD-1, PD-L1, and CTLA-4; targeting either the PD-1 or PD-L1 checkpoint protein; or regulating both transcription and related proteins simultaneously within the TME [69]. For PD-L1 checkpoint molecule expression, since the mechanism of PD-L1 overexpression that results in cancer immune evasion is the disruption of the PD-L1 3′-UTR in several types of cancer, multiple miRNAs bind to the 3′-UTR of PD-L1, resulting in translation inhibition at the posttranscriptional level. As an inhibitor of PD-L1 expression, miR-570 commonly exhibits downregulated expression during HCC oncogenesis, while miR-570 mimics can exert antiproliferative and antiangiogenic effects in vivo [70, 71]*.* In addition, in PD-L1 regulation, miRNAs can work either directly by targeting the 3′-UTR of PD-L1 as mentioned above or indirectly by regulating the upstream pathways of PD-L1, such as the PTEN/PI3K/Akt and JAK/STAT pathway [72–74]. Therefore, targeting the subset of immunomodulatory miRNAs, including miR-34, miR-126, miR-155, and the miR-25-93-106b cluster, and dysregulated miRNAs including miR-34, miR-570, miR-20b, miR-21b and miR-130b to regulate the expression of PD-L1 and control tumor immune evasion would be a promising approach to restore antitumor immunity and enhance the therapeutic response in HCC [71, 73, 75–77]. Since the liver contains the largest population of macrophages (also known as KCs) in the human body, TAMs, especially those with an M2 phenotype, acting as protumor macrophages within the TME of HCC have been “tamed” to facilitate tumor initiation, progression, and metastasis [78]. We summarize the recently proven regulatory approaches that can switch macrophage phenotypes from M2 to M1 in Table 1, including modulatory miRNA methods, immune checkpoint blockade, and other feasible approaches.

**Table 1:** The verified regulating methods and targets that could repolarize TAMs to anti-HCC phenotypes

Phenotypic Change	Regulating method	HCC model	Pathway	Effects
M2 to M1	miR-99b transfection	Hepa1–6 cells injected mice	κB-Ras2 and/or mTOR; a positive feedback regulation loop of NF-κB	miR-99b amplifies M1 macrophage function, resulting in increased phagocytosis and antigen presentation, which impedes the growth of murine HCC [79]
M0 to M1; M2 to M1	a nanoliposome-loaded C6-ceremide (LipC6) injection	C57BL/6 mice received injections of oncogenic hepatocytes	ROS signaling	LipC6 enhances M1 cytokine production while inhibiting M2 cytokine production, thereby reversing immune suppression, and increasing CD8^+^ T cells activity [80]
M0 to M1; M2 to M1	Listeria-based HCC vaccine, Lmdd-MPFG combined with PD-1 blockade	Hepa1–6/MPFG tumor-bearing mice	NF-κB pathway through the TLR2 and MyD88 pathway	Lmdd-MPFG induces an increase in T cells number in the HCC TME and promotes the production of cytokines, such as IFN-γ [81].
M1-type activated; M2-type decreased	dual anti-PD-1/VEGFR-2 therapy	HCA-1 in C3H mice and RIL-175 in C57Bl/6 mice.	selectively upregulated pathways associated with myeloid cells and B cells	Combination PD-1and VEGFR-2 blockade therapy shifts the TAM ratios of F4/80^+^ CD80^+^ and/or CD86^+^ M1 TAMs to F4/80^+^CD206^+^ M2 TAMs, which also regulates the infiltration of other immune cells and reprograms the TME to an antitumor state in HCC [82].
M0 to M1	upregulate RIG-I expression	H22 liver cancer cells inoculated C57BL/6 mice	RIG-I/ MAVS/TRAF2/NF-κB pathway	RIGI-induced M1 macrophages promoted apoptosis and death in HCC cells in vivo and in vitro [83].
M0 to M1	upregulated SIRT1	HepG2 and RAW 264.7, HL-60 macrophages	NF-κB pathway	SIRT1 overexpression enhances M1-like macrophage infiltration in HCC while inhibiting HCC cell growth, migration, and invasion [84].
M2 to M1	upregulate IL-37 expression	HepG2 and Huh-7 cells injected BALB/c nude mice and HCC-conditioned TAMs	IL-6/STAT3 signaling.	IL-37 inhibits M2 polarization of TAMs and then suppresses HCC cell activities, including growth, migration, and invasion. In vivo, upregulated IL-37 expression in HCC-conditioned TAMs delays tumor growth [85].
M2 to M1	miR-98 mimics	HepG2 and SMMC7721 cells incubated with the culture medium of TAMs	targeting IL-10	miR-98 reverses M2 polarization in HCC and inhibits the TAM-mediated promotion of invasion, migration, and epithelial-mesenchymal transition in HCC [86, 87].

In addition, the novel inhibition approaches for the aforementioned Tim-3 checkpoint may provide new ideas for the regulation of other immune checkpoints. In the tumor immune microenvironment, cytokines are connected with immune cell activation and involved in the induction of Tim-3 expression in immune cells. It has been reported that the transcriptional induction of Tim-3 in T cells can be activated by IL-2, IL-7, IL-15, IL-21, and IL-27 [88, 89]. Increased serum IL-6 levels enhance PD- L1 expression in HCC monocytes and macrophages through JAK2/STAT1 and JAK2/STAT3/c- MYC signaling activation [90]. Specifically, Xie et al. analyzed immunohistochemistry and RNA-sequencing data for patients with HCC and demonstrated that high expression of OX40 regulated the activation of T cells, expansion of suppressive Tregs and upregulation of Tim-3 expression as well as CD8A, CD68, LAG3, and PD-1 expression [91], which provided a rationale for reversing multiple immunosuppressive pathways via therapeutic targeting of OX40 and blocking associated immune checkpoints in HCC patients. In addition to checkpoint molecule expression regulation, a new form of blockade now under preclinical scrutiny is also a therapeutic option. Similar to the recognition function of monoclonal antibodies, aptamers, single-stranded or peptide molecules, can bind to specific target molecules and display activity superior to that of currently used RMT3–23 monoclonal antibodies both in vivo and in vitro. Researchers have identified that a trimeric ligand of Tim-3 can efficiently eliminate the interaction of Tim-3 with Gal-9, thereby enhancing the proliferation of Tim-3^+^T cells and promoting their antitumor cytokine secretion with high affinity and specificity [92]. For Tim-3-expressing lymphocytes, Hervas-Stubbs et al. isolated a Tim-3 nonantigenic oligonucleotide aptamer (Tim-3 Apt) that binds to the extracellular domain of Tim-3 and increase the secretion of IFN-γ [93]. Moreover, MP7, an aptamer that blocks the immunoinhibitory PD-1, functionally restores IL-2 secretion in primary T cells by inhibiting the interaction between PD-1 and PD-L1 [94]. Since aptamers have potency equivalent to that of an antagonistic anti-checkpoint molecule antibody that provokes robust and durable antioncogenetic responses and inherent advantages, including a lack of immunogenicity, low cost, long shelf life, and antidote availability, the development of aptamers as novel ICBs for patients with HCC has bright research prospects.

## Discussion

Tumor growth and metastasis are related not only to the intrinsic characteristics of tumor cells but also to the organ microenvironment where tumorigenesis is occurring, which is the dominant force promoting tumor growth [95]. The TME is recognized as a highly dynamic network during the occurrence, development, and prognosis of cancer or therapeutic interventions and one of the leading causes of tumor heterogeneity. The TME is composed of immune cells and stromal cells, the two major nontumor components that largely determine the effect and prognosis of cancer treatment [96]. Currently, to transform the TME into an immunologically “hot” status, novel methods that combine metabolic regulation and microbiome-based gene signatures that facilitate personalized immunotherapies are urgently needed to enhance antitumor immunity. Riera-Domingo et al. [97] emphasized hypoxic signatures that influence the immune response to immunotherapy. The hypoxic microenvironment has a great impact on the antitumor immune response. As a critical process that evolves in the TME, hypoxia affects epithelial-mesenchymal transition and angiogenesis and mainly changes the expression of immune checkpoint molecules, such as PD-L1, CD47, PD-1, and HLA-G, in liver cancer [98, 99]. With the help of HIF-1α, which binds the HRE in the promoter of the PD-L1 gene, the expression of PD-L1 is significantly increased in many types of tumor cells, including melanoma, lung cancer, breast cancer, and prostate cancer cells [100, 101]. In the hypoxic microenvironment of a steatotic-HCC mouse model, Jianxu et al. reported that upregulated HIF-2α expression led to lipid accumulation through PI3K-AKT-mTOR pathway activation, which reflected that the hypoxic conditions with HIF-2α upregulation could serve as a potential therapeutic target for liver cancer treatment [102]. Interestingly, there are always highly hypoxic conditions within the TME that promote immunosuppression and negatively affect antitumor immunity, yet one recent study found that CD8^+^ T cells activated under hypoxic condition exhibited increased cytotoxicity and enhanced antitumor immunity in vivo [103]. Therefore, approaches for regulating microenvironmental hypoxia in HCC should be given more attention in future studies, including direct modulation of HIF-mRNA, induction of HIF-1α degradation, hypoxia-activated drugs, small-molecule HIF inhibitors, and drugs targeting signaling pathways downstream of HIF [104, 105].

In addition to regulation of the microenvironment at the metabolic level, targeting the regulation of immune cells seems to be relatively feasible. As mentioned above, studies have consistently verified the potential of TAMs as a target for HCC immunotherapy. Therefore, the development of better tools, such as high-throughput single-cell approaches, could allow in-depth analysis of the phenotypic characteristics and functional features of diverse cell types within the TME and reveal their crosstalk at different stages of cancer progression and metastasis [10], thereby guiding clinical therapeutics for tumors and prognostic monitoring. Since the two major classes of tumor-associated macrophages have distinct opposing roles in the development of HCC, inducing the transition from protumor M2-like macrophage phenotype to a tumoricidal M1 phenotype is the main strategy to increase the antitumor effect of TAMs. Therefore, we also summarized the emerging potential regulatory methods that could achieve this transformation in Table 2. Furthermore, interfering with M2- like TAM survival and blocking M2 macrophage recruitment [113] are also two targeted regulatory mechanisms that cannot be ignored.

**Table 2:** The potential targets and pathways that regulate HCC-TAMs phenotypic transition

Target	Pathway	Cell line or HCC model	Pro-tumor Results
RIPK3 and FAO	ROS-caspase1-PPAR pathway	Murine H22 cells and C57BL/6 WT mice injected with diethylnitrosamine as HCC model	The RIPK3-FAO-ROS-caspase1-PPAR signaling axis is responsible for increased M2-TAM infiltration, which promotes HCC tumorigenesis [106].
LINC00662	WNT3A-Wnt/b-catenin signaling	HCCLM3, MHCC97H, Huh7, SK-HEP-1, and Hepa1–6 HCC cells	By upregulating WNT3A expression, LINC00662 activates the Wnt/b-catenin pathway and then induces M2 macrophage polarization, contributing to HCC tumorigenesis and invasion and repressing HCC cell apoptosis [107, 108].
IL-25	**/**	Human HCC cell lines MHCC97L and HepG2 cells; Murine HCC cell lines H22, and Hepa1–6; and BALB/c nude mice with the portal venous injection macrophages as HCC model.	IL-25 facilitates M2 TAM (CD206/CD68) infiltration, promotes secretion of the chemokine CXCL-10, and then induces HCC progression through the EMT pathway [109].
CAF-induced endosialin	Interaction with CD68 and regulate GAS6 expression in CAF	Huh7 cell HFL-1 cells	In CAFs, via an interaction with CD68, endosialin promotes the expression of GSA6, which results in increased M2 macrophages recruitment and HCC progression [110].
HCC-derived HMGB1	ROS-TLR2-NOX2-autophagy axis	Mouse hepatoma cell line ML-14a cells and murine in a situ hepatoma model	Hepatoma-derived HMGB1 stimulates ROS via the TLR2/NOX2 axis, thereby inducing M2 macrophage polarization and subsequently supporting HCC growth [111].
Nogo-B	Nogo-B-Yap/Taz pathway	Murine HCC cell lines Hepa1–6 cells and H22 cells, macrophage cell line RAW 264.7 cells	Elevated Nogo-B expression facilitates M2 TAM polarization and promotes HCC tumor growth in vivo by inducing Yap/Taz signaling [112].

## Conclusion

In conclusion, as the liver is the body’s largest immune organ, liver cancer occurrence and development are already complicated, which results in the TME of liver cancer constantly changing. Nevertheless, modulation of the highly immunosuppressive and metabolically stressed TME is urgently needed to improve efficacy and identify protagonist cell types, such as TAMs, with high therapeutic relevance in the TME of both primary HCC and metastatic HCC. Furthermore, immune checkpoint inhibition has been studied in detail to explore the relevant mechanisms of PD-1/PD-L1 and CTLA-4, but specific useful ICB targets have not produced reflected a relatively ideal effect in HCC treatment. Additionally, recent works in the literature have recognized the role of Tim-3 as a potential target in HCC immunotherapy, which might both enhance T cell-induced antitumor immunity and revise macrophage-mediated immunosuppression in the HCC microenvironment, suggesting that future immune checkpoint investigations may focus more on Tim-3 for treatment of HCC or other solid tumors with similar pathogenesis. In addition to novel immune checkpoint discovery, emerging regulatory strategies have inspired HCC immunotherapy ideas. Immunotherapeutic aptamers, which bind specifically to the extracellular domain of immune checkpoints and block their interactions with receptors/ligands, significantly suppress growing tumor cells in vivo and represent an attractive alternative to monoclonal antibody therapeutics. Importantly, these antagonistic aptamer treatments have important advantages, including lower antigenicity, lower manufacturing price, and higher malleability. Additionally, at the molecular level of regulation, recent discoveries have shown that some miRNAs and hypoxia can negatively affect the antitumor immune response by modifying the expression of the main immune checkpoint molecules, setting up new therapeutic intervention opportunities to enhance the clinical benefit derived from ICBs. In the future, much attention should be paid to altering systemic metabolism or upstream modulatory sites in multiple facets of antitumor immunity within the HCC microenvironment, thereby significantly increasing the sensitivity of immune surveillance and enhancing treatment responses.

## Data Availability

Data sharing is not applicable to this article as no datasets were generated or analyzed during the current study.

## Abbreviations

HCC: Hepatocellular carcinoma
HBV: Hepatitis B virus
HCV: Hepatitis C virus
TME: Tumor microenvironment
ECM: Extracellular matrix
TIME: Tumor immune microenvironment
MDSCs: Myeloid-derived suppressor cells
Treg: Regulatory T
TAMs: Tumor-associated macrophages
NKs: Natural killer cells
I-E: Infiltrated-excluded
I-I: Infiltrated-inflamed
TLS: Tertiary lymphoid structure
CTLs: Cytotoxic lymphocytes
PD-1: Programmed cell death 1
PD-L1: PD-1 ligand
RFA: Radiofrequency ablation
TACE: Trans arterial chemoembolization
TARE: Trans arterial radioembolization
VEGF: Vascular endothelial growth factor
TKI: Tyrosine kinase inhibitor
PDGFR: Platelet-derived growth factor
FGFR: Fibroblast growth factor
ICB: Immune checkpoint blockade
CSF: Colony-stimulating factor
iCCA: Intrahepatic cholangiocarcinoma
IFN: Interferon
LGDNs: Low-grade dysplastic nodules
HGDNs: High-grade dysplastic nodules
TIL: Tumor-infiltrating lymphocyte
APCs: Antigen-presenting cells
KCs: Kupffer cells
TLRs: Toll-like receptors
PGE2: Prostaglandin E2
TCGA: The Cancer Genome Atlas
Tim-3: Immunoglobulin mucin-3
TGF-β: Transforming growth factor-β
ICI: Immune checkpoint inhibition
CTLA-4: Cytotoxic T-lymphocyte antigen 4
IDO: Indoleamine 2,3-dioxygenase
LAG-3: Lymphocyte activation gene 3
TAA: Tumor-associated antigen
Tim-3 Apt: Tim-3 nonantigenic oligonucleotide aptamer
